# Encouragement of cervical cancer screening via an evolutionary theoretical approach: A randomized controlled study in Japan

**DOI:** 10.1016/j.pmedr.2022.101818

**Published:** 2022-05-10

**Authors:** Tsuyoshi Okuhara, Hiroko Okada, Eiko Goto, Aiko Tsunezumi, Yumi Kagawa, Takahiro Kiuchi

**Affiliations:** Department of Health Communication, School of Public Health, The University of Tokyo, Tokyo, Japan

**Keywords:** Cervical cancer, Cancer screening, Behavior change, Evolutionary psychology, Health communication

## Abstract

The introduction of an evolutionary perspective into public health research has received attention in recent years. We aimed to examine the effects of messages that target the fundamental human motive of kin care (i.e., childbirth and parenting) on cervical cancer screening recommendations, based on an evolutionary theoretical approach. A randomized controlled study was conducted in Japan. Female participants (n = 969) were randomly assigned either to a group that received an intervention message that targeted the fundamental motive of kin care (recommending cervical cancer screening for future childbearing), or that targeted the fundamental motive of disease avoidance, or a control message. Intention to obtain cervical cancer screening was assessed both before and after reading the messages. A one-way ANOVA with Tukey’s or Games–Howell test was conducted. Each of the intervention message targeting the fundamental motive of kin care and of the message targeting disease avoidance significantly increased intention to obtain cervical cancer screening versus a control message (M = 0.76 vs. M = 0.17, *p* < 0.001; M = 0.74 vs. M = 0.17, *p* < 0.001, respectively). A message that targeted the fundamental motive of kin care was as effective as one targeting the fundamental motive of disease avoidance. Health professionals should add messages that target the fundamental motive of kin care to their repertoire to encourage cervical cancer screening among women who wish future childbirth and parenting (e.g., “Delayed detection of cervical cancer may prevent your future childbirth and parenting. So let’s obtain cervical cancer screening regularly for your future childbirth and parenting.”)

## Highlights

• This is the first study of evolutionary approaches to encourage cancer screening.
• We tested the effect of a message targeting the fundamental human motive of kin care.
• Kin care messages are such as “get cervical cancer screening for future childbirth.”
• A message targeting the fundamental motive of disease avoidance was also tested.
• The kin care message was as effective as the disease avoidance message.

## 1 Introduction

Cervical cancer is the fourth most common cancer in women, with over 570,000 new cases a year (World Health Organization, 2020). Cervical cancer is curable if detected early at pre-cancer stages by cancer screening and adequately treated (Landy et al., 2016). In November 2020, WHO announced the “Global strategy to accelerate the elimination of cervical cancer as a public health problem” (World Health Organization, 2020). It has suggested screening 70 percent of women for cervical cancer at ages 35 and 45 (World Health Organization, 2020). However, cervical cancer screening coverage is inequitably distributed across geographical settings and income. Although higher income countries tend to achieve higher screening coverage, coverage remains low in some high-income countries such as 30% in Hungary, 39% in Italy, 31% in Turkey, and 44% in Japan in 2019 (percentage of females aged 20–69 screened) (OECD Statistics, 2021). Further efforts are needed to develop more effective communication strategy to encourage to obtain cervical cancer screening.

Various models and theories have been used in studies of methods to encourage to obtain cervical cancer screening, such as the health belief model, protection motivation theory, and theory of planned behavior (Musa et al., 2017; Wollancho et al., 2020; Calderón-Mora et al., 2020). These models and theories emphasize cognitive beliefs about health behaviors, such as perceived susceptibility of getting cervical cancer and perceived severity of cervical cancer. However, the effects of behavioral change resulting from interventions using these theories and models are not as large as has been expected (Conner and Norman, 2017). Existing cognitive behavioral models have been criticized for focusing on proximate causes of cognitive influence on health behaviors at the expense of ultimate causes of human behaviors (Griskevicius and Kenrick, 2013).

In the present study, we adopt another approach grounded in an evolutionary theoretical framework by focusing on fundamental human motives. In recent years, researchers have discussed the introduction of an evolutionary perspective into health behavior research (Eaton et al., 2002, Graham and Martin, 2012), and *The Lancet* published an evolutionary public health series in 2017 (Wells et al., 2017, Jasienska et al., 2017, Rook et al., 2017). Those studies suggest that, in order to understand health behavior, we must connect the various choices we make in our day-to-day lives with their evolutionary meaning. Evolutionary biologists have presumed that all living organisms have been selected to maximize their relative success at passing genes into future generations via either direct reproduction or helping kin reproduce, which they call inclusive fitness (West and Gardner, 2013).

Because humans are a highly social species, they have faced and solved crucial social challenges to enhance their inclusive fitness. Evolutionary psychologists have assumed that these evolutionary challenges include self-protection (protecting oneself from enemies and predators), disease avoidance (avoiding infection and disease), affiliation (forming and maintaining cooperative alliances), status (gaining and maintaining respect and prestige of their fellow members), mate acquisition (successfully attracting and acquiring a romantic partner), mate retention (fostering long-term mating bond with that person), and kin care (investing in and caring for family and kin) (Griskevicius and Kenrick, 2013, Kenrick et al., 2010, Schaller et al., 2017). These seven are the fundamental human motives to enhance inclusive fitness (Griskevicius and Kenrick, 2013, Kenrick et al., 2010, Schaller et al., 2017).

According to the concept of domain specificity, which is one of the key features of modern evolutionary approaches (Clark et al., 2015, Barrett and Kurzban, 2006), a different psychological system guides each decision, depending on which fundamental motive is currently paramount on an individual’s mind (Griskevicius and Kenrick, 2013, Kenrick et al., 2010, Schaller et al., 2017). Each of the seven fundamental motives is assumed to develop in stages as an individual grows through childhood and adolescence to old age, based on the life history theory (del Giudice et al., 2016). However, even as one of the seven motives is paramount, other motives are ready to be activated and become paramount by external or internal cues that indicate threats or opportunities related to a specific evolutionally challenge (Griskevicius and Kenrick, 2013, Kenrick et al., 2010, Griskevicius et al., 2009).

Previous studies have shown that communication of cervical cancer and cancer screening have primarily communicated the benefits of early detection of cancer by screening (Okan et al., 2019, Okuhara et al., 2017). Namely, communication to encouragement of cervical cancer screening to date has often targeted the fundamental motive of disease avoidance (e.g., “Cervical cancer is curable if detected early. Let's obtain a cervical cancer screening”). However, in a safe and hygienic environment such as that of a modern industrialized country, the fundamental motive of disease avoidance may be inert. Additionally, prolonged exposure to similarly-themed messages generates psychological reactance and disengagement toward incoming messages, leading to ineffective persuasive outcomes (Ball and Wozniak, 2021, Kim and So, 2018).

Contrarily, the fundamental motive of kin care can be paramount in the minds of women who wish future childbirth and parenting, although messages targeting this fundamental motive have rarely been used in communication to encourage cervical cancer screening to date (Okan et al., 2019, Okuhara et al., 2017) (e.g., Delayed detection of cervical cancer may prevent your future childbirth and parenting. So let’s obtain cervical cancer screening regularly for your future childbirth and parenting.) Therefore, we hypothesize that a cervical cancer screening recommendation message that targets the fundamental motive of kin care will be equally effective or more effective in encouraging cervical cancer screening among women who wish future childbirth and parenting than a message that targets the fundamental motive of disease avoidance.

However, to our knowledge, no study has focused on the fundamental human motives based on the evolutionary theoretical approach and examined their influence on health decision making including cervical cancer screening. The aim of this study is to examine the persuasive effects of messages that target the fundamental motive of kin care on cervical cancer screening recommendations, and to investigate the usefulness of developing messages to encouragement of cervical cancer screening based on an evolutionary theoretical approach.

## 2 Methods

### 2.1 Participants and design

**Fig. 1:**
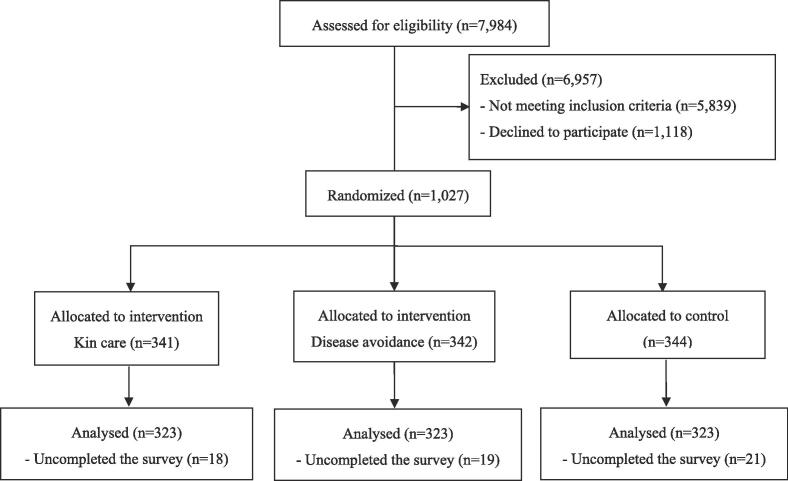
Participant flow diagram.

This was a single blinded, a three group parallel design randomized controlled study conducted in Japan. Participants were recruited from people registered in a survey company database. E-mails were sent to registered users who responded to screening questions. The inclusion criteria were women under the age of 40 who wish to have a baby in the future because fertility declines sharply after the age of 37 (The American College of Obstetricians and Gynecologists Committee on Gynecologic Practice and The Practice Committee of the American Society for Reproductive Medicine), and that even with assisted reproductive technology, the pregnancy and delivery rates decline sharply after age 39 (Japanese Society of Obstetrics and Gynecology ART Data Book, 2019): this is recognized by over 70% of Japanese women (Maeda et al., 2015). Women who already have children and are currently pregnant were excluded because kin care message was targeted at women who wishes to take care of their child in the future. Those who regularly obtain cervical cancer screening were excluded because there is no need to provide additional recommendations to those who voluntarily undergo regular screening. Women who have had cervical cancer themselves or someone close to them have had cervical cancer were also excluded because examples of illness experiences of people close to them influence their health behaviors (Bigsby et al., 2019). Fig. 1 shows the participant flow diagram.

Recipients who were eligible and consented to participate via online were invited to complete a web-based survey. When participants consented to participate in the study via the website, they were randomly assigned with a 1:1 allocation to a group that received either an intervention message that targeted the fundamental motive of kin care, an intervention message that targeted the fundamental motive of disease avoidance, or a control message, using an algorithm included in the survey program. A total of 1027 participants were randomized and 969 participants completed the survey from October 7 to 8, 2021. The methods of the present study adhered to CONSORT guidelines (Schulz et al., 2010). The study was registered as a University Hospital Medical Information Network Clinical Trials Registry Clinical Trial (UMIN000045387) on September 5, 2021. The protocol was approved by the ethical review committee at the Graduate School of Medicine, University of Tokyo (2021155NI). All participants gave written informed consent in accordance with the Declaration of Helsinki.

### 2.2 Intervention and control messages

Appendix A1 shows the intervention and control messages, translated into English for this report. We created intervention messages that targeted the fundamental motive of kin care and that targeted the fundamental motive of disease avoidance by referring to information from the Ministry of Health, Labor and Welfare. To appeal to the fundamental motive of kin care, the message conveyed that delayed detection of cervical cancer has a negative impact on pregnancy and childbirth. Participants in the intervention group that received a message that targeted the fundamental motive of kin care read a short message (Appendix A2) and viewed a short video about parenting, just prior to reading the intervention message. These comment and video were used to activate the fundamental motive of kin care.

As mentioned earlier, existing cervical cancer screening recommendation messages have primarily communicated content of disease avoidance. We created the intervention disease avoidance message that mimics typical existing cervical cancer screening recommendation messages in Japan. To appeal to the fundamental motive of disease avoidance, the message conveyed that delayed detection leads to death or sequelae and early detection leads to high survival rates.

In this study, in order to detect the effect of the message using an evolutionary approach by comparing the intervention and control groups, the control message had to be content that did not affect the outcome, i.e., content that was not related to cervical cancer. Therefore, for a control message we obtained textual information about bruxism from the website of the Ministry of Health, Labour and Welfare (https://www.e-healthnet.mhlw.go.jp/).

### 2.3 Measures

The primary outcome was intention to obtain cervical cancer screening. Participants responded to the following four questions on a scale of 1 to 6, ranging from “extremely unlikely” to “unlikely,” “a little unlikely,” “a little likely,” “likely,” and “extremely likely”: (1) I will make sure to obtain a cervical cancer screening every two years. (2) If I could make an appointment for a cervical cancer screening right now, I would do it right now. (3) Even if I am busy, I will make time to obtain a cervical cancer screening. (4) Even if I am afraid to obtain a cervical cancer screening, I will do it. We adapted this measure from previous studies (Chan et al., 2011, Okuhara et al., 2018).

The secondary outcome was attitude toward obtaining cervical cancer screening. Participants rated “obtaining cervical cancer screening” on a scale consisting of six 1–6 semantic differential items (bad/not bad, beneficial/not beneficial, harmful/not harmful, good/not good, valuable/not valuable, and important/not important). This measure was adapted from previous studies (Dormandy et al., 2006, Hersch et al., 2014).

Participants also responded to the following questions on a scale of 1 to 6 as mentioned above: (1) Having and raising my own child is my greatest motive among my various motives. (2) Not getting sick is my greatest motive among my various motives. Each of these two questions were originally developed by the authors. They served as a manipulation check to examine if each intervention message activated the fundamental motive of kin care and disease avoidance, respectively.

All these questions were measured before and after the participants read intervention or control messages. Mean scores (ranged from 1 to 6) were used for the analysis. Higher scores indicate greater intentions, more favorable attitudes, and greater motives. Additionally, all participants were asked for their sociodemographic information before they read intervention or control messages.

### 2.4 Sample size

Based on the effect size in previous randomized controlled studies (Hopfer, 2012, Okuhara et al., 2020), we estimated a small effect size (f = 0.10) in the present study. We conducted a power analysis at an alpha error rate of 0.05 (two-tailed) and a beta error rate of 0.20. The power analysis indicated that 323 participants were required in each of the intervention and control groups.

### 2.5 Statistical analysis

Descriptive statistics were used to summarize participants’ sociodemographic information as percentages for categorical variables and as mean ± standard deviation for continuous variables. Cronbach’s α values were used to determine the internal reliability of the outcome measures. A one-way analysis of variance (ANOVA) was conducted with the absolute change in mean values before and after intervention and the mean values after intervention for each measure as the dependent variable and the group assignment as the independent variable. For multiple comparisons, Tukey’s test was conducted on significant main effects where appropriate. The Games–Howell test was performed when the assumption of homogeneity of variances was not satisfied. A p value of <0.05 was considered significant in all statistical tests. All statistical analyses were performed using IBM SPSS Statistics for Windows, Version 21.0 (IBM, Armonk, NY, USA).

## 3 Results

### 3.1 Participant characteristics

Table 1 shows the participants’ characteristics. Participant age ranged from 20 to 39 years (mean = 28 years, SD = 5.1). 59% of participants had an educational attainment level beyond university graduation. 29% of participants had an annual household income of six million yen or more (one US dollar is roughly equivalent to 100 yen). Participants were distributed throughout Japan.

**Table 1:** Participants’ characteristics.

	Kin care (n = 323)	Disease avoidance (n = 323)	Control (n = 323)	Total (n = 969)
**Age, mean years (SD)**	27.6 (5.0)	27.5 (5.0)	27.7 (5.1)	27.6 (5.1)
**Highest education, %**				
Less than high school	1.2	0.6	0.3	0.7
High school graduate	20.7	16.1	19.5	18.8
Some college	16.4	23.8	22.9	21.1
University graduate	56.3	54.8	52.9	54.7
Graduate school	5.0	3.7	4.0	4.2
Others	0.3	0.9	0.3	0.5
**Household income, %**				
Less than 2 million yen^a^	14.6	15.8	14.9	15.1
2–4 million yen	31.3	34.7	33.7	33.2
4–6 million yen	26.3	21.1	21.7	23.0
6–8 million yen	15.2	13.0	15.2	14.4
8–10 million yen	6.2	8.0	5.0	6.4
More than 10 million yen	6.5	7.4	9.6	7.8

a One US dollar is roughly equivalent to 100 yen.

### 3.2 Comparison of outcomes between groups

Cronbach’s α for internal consistencies of questions were 0.915 in intention to obtain cervical cancer screening and 0.878 in attitude toward obtaining cervical cancer screening.

Table 2 shows the intention of and attitude toward obtaining cervical cancer screening across groups. Regarding the absolute change in mean values for intention to obtain cervical cancer screening before and after the intervention, ANOVA revealed a significant main effect of the group assignment [*F*(2, 966) = 69.286, *p* < 0.001, η^2^ = 0.125]. The Games–Howell *post hoc* test revealed significant differences between the kin care and the control groups (M = 0.76 vs. M = 0.17, *p* < 0.001), and the disease avoidance and the control groups (M = 0.74 vs. M = 0.17, *p* < 0.001). There was no significant difference between the two intervention groups. Regarding the mean values for intention of obtaining cervical cancer screening after the intervention, ANOVA and multiple comparisons revealed the same trend as the absolute change. Regarding the absolute change in mean values for attitude toward obtaining cervical cancer screening before and after the intervention, ANOVA and multiple comparisons revealed the same trend as the intention.

**Table 2:** Comparisons of measures between groups.

		Kin care (n = 323)	Disease avoidance (n = 323)	Control (n = 323)	*p*^d^
Intention	Before	3.62 a (0.99) b	3.65 (1.06)	3.55 (1.01)	–
	After	4.40 (0.98)*	4.39 (1.07)**	3.72 (1.06)	< 0.001
	Change	**0.76 (0.69–0.86)**^c^**	0.74 (0.65–0.82) **	0.17 (0.09–0.24)	< 0.001
Attitude	Before	5.12 (0.88)	5.14 (0.90)	5.01 (0.90)	–
	After	5.39 (0.88)**	5.41 (0.85)**	5.08 (0.92)	< 0.001
	Change	0.26 (0.19–0.33)**	0.27 (0.20–0.33)**	0.06 (0.01–0.11)	< 0.001
Motive of kin care	Before	4.15 (1.28)	4.14 (1.28)	4.10 (1.17)	–
	After	4.49 (1.28)*	4.36 (1.35)	4.23 (1.24)	0.04
	Change	0.33 (0.24–0.42)*	0.22 (0.15–0.29)	0.13 (0.06–0.19)	0.001
Motive of disease avoidance	Before	4.53 (1.01)	4.57 (1.11)	4.53 (1.01)	–
	After	4.84 (1.10)*	4.88 (1.13)*	4.63 (1.04)	0.01
	Change	0.31 (0.23–0.39)**	0.30 (0.23–0.39)**	0.10 (0.03–0.16)	< 0.001

*Significantly higher than the control group by multiple comparisons (*p* = 0.05).

**Significantly higher than the control group by multiple comparisons (*p* < 0.001).

a Mean.

b Standard deviation.

c 95% confidence interval.

d *p* values for comparing amount of change among groups using ANOVA.

### 3.3 Manipulation check

As Table 2 shows, for absolute change in mean values for the motive of kin care before and after the intervention, ANOVA revealed a significant main effect of the group assignment [*F*(2, 966) = 6.968, *p* = 0.001]. The motive of kin care increased the most in the intervention group that targeted the fundamental motive of kin care; the Games–Howell post hoc test revealed a significant difference between the kin care and the control groups (M = 0.33 vs. M = 0.13, *p* = 0.001). The absolute change for motive of kin care in the intervention group that targeted the fundamental motive of kin care was higher than in the intervention group that targeted the fundamental motive of disease avoidance (M = 0.33 vs. M = 0.22), although the difference was not significant.

In terms of absolute change in mean values for the motive of disease avoidance before and after the intervention, ANOVA revealed a significant main effect of the group assignment [*F*(2, 966) = 10.050, *p* < 0.001]. The Games–Howell *post hoc* test revealed a significant difference between the kin care and the control groups (M = 0.31 vs. M = 0.10, *p* < 0.001), and the disease avoidance and the control groups (M = 0.30 vs. M = 0.10, *p* < 0.001).

## 4 Discussion

We conducted interventions to examine the effectiveness of a cervical cancer screening recommendation message that targeted the fundamental human motive of kin care of women who wish future childbirth and parenting, based on the evolutionary theoretical approach. A message that targeted the fundamental motive of kin care significantly improved attitudes toward cervical cancer screening and significantly increased intention to obtain cervical cancer screening compared to a control message. Previous research and practice on behavior change, including cervical cancer screening recommendations, have been based mainly on cognitive behavioral models, and focused on cognitive beliefs such as perceived susceptibility and perceived severity of getting cervical cancer (Musa et al., 2017, Wollancho et al., 2020, Calderón-Mora et al., 2020). Previous studies have shown that many cervical cancer screening recommendation messages target the fundamental motive of disease avoidance and communicate the benefits of early detection of cancer by screening (Okan et al., 2019, Okuhara et al., 2017). However, repeated exposure to messages with similar themes generates a psychological reactance and disengagement toward incoming messages, leading to ineffective persuasive outcomes (Ball and Wozniak, 2021, Kim and So, 2018). Therefore, the results of this study imply that health professionals should deliver messages that target not only the fundamental motive of disease avoidance, but also the fundamental motive of kin care, to increase the effectiveness of communication to encourage to obtain cervical cancer screening toward women who wish future childbirth and parenting.

This communication strategy targets the fundamental motive of kin care may bring a new option to health professionals who experience difficulty in communicating about cervical cancer screening. First, cancer screening information requires a certain level of literacy and numeracy to understand, making it difficult for those with low health literacy to understand and use cancer screening information (Kim and Han, 2016). However, messages that target the fundamental motive of kin care are simple and straightforward and may influence people of all health literacy levels (Jun, 2020). Second, health organizations and professionals are recommended to deliver effective tailored messages to encourage cervical cancer screening (Fornos et al., 2014). Messages that target the fundamental motive of kin care can be used to deliver tailored personally relevant messages to those who wish future childbirth and parenting. Third, messages that target the fundamental motive of kin care can be used in multiple channels—which are also recommended for effective cancer screening communication—such as online, offline and face-to-face communication (Ravindran et al., 2020, Walsh-Childers et al., 2018).

A message that targeted the fundamental motive of disease avoidance also significantly improved attitudes toward cervical cancer screening and significantly increased intention to obtain cervical cancer screening compared to a control message. These results indicate that a message that targets the fundamental motive of disease avoidance also encourages to obtain cervical cancer screening among women who wish future childbirth and parenting. Cancer screening recommendation messages to date have mainly targeted the fundamental motive of disease avoidance (Okan et al., 2019, Okuhara et al., 2017); the results of this study indicate that these cancer screening recommendation messages have had some effect in encouraging cervical cancer screening. Therefore, the results of this study imply that health professionals should continue to deliver messages that target the fundamental motive of disease avoidance to encourage cervical cancer screening.

The present study found no significant difference in effectiveness between the two intervention messages; namely, a message that targeted the fundamental motive of kin care was as effective as a message that targeted the fundamental motive of disease avoidance for encouraging cervical cancer screening. This result indicates that previous strategies for communicating cervical cancer screening recommendations, which primarily used messages that targeted the fundamental motive of disease avoidance, were not the only optimal choice. As noted above, it is recommended that health professionals add messages that target the fundamental motive of kin care to their repertoire of cervical cancer screening recommendations.

Future studies should examine the effect of messages that target the fundamental motive of kin care on encouraging health behavior change other than cervical cancer screening. For example, messages that target the fundamental motive of kin care may encourage adolescent girls to receive HPV vaccination (e.g., Cervical cancer may prevent your future childbirth and parenting. So let’s receive HPV vaccination for your future childbirth and parenting). Additionally, there are other fundamental human motives of such as affiliation, status, mate acquisition, and mate retention than kin care. Future studies should examine the effectiveness of public health messages that target these fundamental motives.

The present study has several limitations. First, this study assessed intentions directly after message exposure; future studies should examine the long-term effects of messages because cervical cancer screening needs to be obtained regularly. Second, this study assessed intentions to obtain cervical cancer screening rather than actual behaviors because intention is easily measurable proxy outcomes for behavior and intention-behavior consistency has been reported (Webb and Sheeran, 2006): however, the gap between intention and behavior should be noticed. Finally, participants in this study were women with no children under the age of 40 who wish to have a first baby in the future. It is unclear as to what extent the present findings are generalizable to populations other than the participants in this web-based study.

## 5 Conclusion

This is the first study to examine the effectiveness of cervical cancer screening recommendation messages by focusing on the fundamental human motives based on the evolutionary theoretical approach. We found that a message that targeted the fundamental human motive of kin care—which has been rarely used—was as effective in encouraging cervical cancer screening among women who wish future childbirth and parenting as a message that targeted the fundamental human motive of disease avoidance, which has been frequently used in cognitive behavioral models. This result indicates that the evolutionary theoretical approach that focuses on fundamental human motives has the potential to extend the communication strategy for cervical cancer screening recommendations. Health professionals should add messages that target the fundamental motive of kin care to their repertoire to encourage cervical cancer screening among women who wish future childbirth and parenting. Messages that target the fundamental motive of kin care are those such as, “Delayed detection of cervical cancer may prevent your future childbirth and parenting. So let’s obtain cervical cancer screening regularly for your future childbirth and parenting.”

## Funding

This work was supported by the Japan Society for the Promotion of Science KAKENHI (grant number 19K10615).

### CRediT authorship contribution statement

**Tsuyoshi Okuhara:** Conceptualization, Methodology, Data curation, Formal analysis, Writing – original draft, Writing – review & editing, Funding acquisition. **Hiroko Okada:** Writing – review & editing. **Eiko Goto:** Writing – review & editing. **Aiko Tsunezumi:** Writing – review & editing. **Yumi Kagawa:** Writing – review & editing. **Takahiro Kiuchi:** Writing – review & editing, Supervision.

## Declaration of Competing Interest

The authors declare that they have no known competing financial interests or personal relationships that could have appeared to influence the work reported in this paper.
