# Supplementary material for: Encouragement of cervical cancer screening via an evolutionary theoretical approach: A randomized controlled study in Japan
Source: Prev Med Rep. 2022 May 10;27:101818. doi: 10.1016/j.pmedr.2022.101818 (PMC9152791; doi:10.1016/j.pmedr.2022.101818)
Supplement: Supplementary data 1 [file mmc1.docx]

**Appendix 1.** Intervention messages used in this study, translated into English

**An intervention message that targeted the fundamental motive of kin care**

Cervical cancer is the most common cancer among women in their 20s and 30s.

Since 2000, the number of cases and the mortality rate have been increasing.

Cervical cancer has almost no early symptoms.

If detected too late, the woman may have to undergo surgery to remove the uterus, which will prevent her from becoming pregnant and giving birth.

Even if you are able to conceive, there is a higher chance of premature birth or low birth weight babies.

Therefore, early detection through medical checkups is very important.

If detected early, the uterus can be preserved and pregnancy and childbirth are possible.

For the sake of future pregnancy and childbirth, women over 20 years old should have a cervical cancer screening every two years.

The examination takes about 15 minutes. There is almost no pain.

**An intervention message that targeted the fundamental motive of disease avoidance**

Cervical cancer is the most common cancer among women in their 20s and 30s.

Since 2000, the number of cases and the mortality rate have been increasing.

Cervical cancer has almost no early symptoms. Early detection through medical checkups is important.

If detected too late, 4 out of 5 women will die (5-year survival rate: 22%).

If it is detected early, about 9 out of 10 people will live (5-year survival rate: 93%).

Treatment after the disease has progressed may result in sequelae such as difficulty in defecation and urination and lymphedema.

Women over the age of 20 should undergo a cervical cancer screening every two years.

The examination takes about 15 minutes. There is almost no pain.

**A control message**

According to the traditional definition, grinding one’s teeth is when somebody makes a sound by strongly grinding the teeth together, usually unconsciously or while asleep. Nowadays, it is often referred to as ‘teeth grinding,’ a term which also covers various actions that we do whilst awake.

Whether you are sleeping or awake, the non-functional biting habit of grinding one’s teeth dynamically or statically, or clenching one’s teeth, can also be referred to as bruxism (sleep bruxism if it occurs at night). Bruxism can be categorized into the movements of: sliding the upper and lower teeth together like mortar and pestle (grinding); firmly and statically engaging the upper and lower teeth (clenching); and dynamically bringing the upper and lower teeth together with a tap (tapping).

Bruxism is difficult to diagnose, as it often has no noticeable symptoms. Stress and dentition are thought to be causes of bruxism, but it is currently unclear and future re-search is anticipated.

**Appendix 2.** A comment that was presented prior to the intervention message that targeted the fundamental motive of kin care (translated into English)

The following is a comment from a woman who is raising her child.

“Giving birth was the single best encounter I’ve ever had in my life.

My world has changed since I had a baby.

When I see the smile on my child’s face, I love her so much that I feel a pang in my heart.

When I see a child’s smile, I feel so moved that I almost cry.

Just stroking the fluffy hair of my child makes me happy.

I’m happy just to feel the touch of her hand on my face.

I’m happy to be at the center of my child’s world.

For the first time, I had an existence that was more important than my own life.

After having a child, I understood the true meaning of happiness.”
